# *Borrelia burgdorferi* sensu lato in *Ixodes ricinus* ticks from Norway: evaluation of a PCR test targeting the chromosomal *flaB* gene

**DOI:** 10.1007/s10493-012-9585-2

**Published:** 2012-06-09

**Authors:** Andrew Jenkins, Dag Hvidsten, Andreas Matussek, Per-Eric Lindgren, Snorre Stuen, Bjørn-Erik Kristiansen

**Affiliations:** 1 Unilabs Telelab, Skien, Norway; 2 Department of Microbiology and Infection Control, University Hospital of North Norway, Tromsø, Norway; 3 Clinical Microbiology Laboratory, Division of Medical Services, Department of Laboratory Medicine, County Hospital Ryhov, 55185 Jönköping, Sweden; 4 Division of Medical Microbiology, Department of Clinical and Experimental Medicine, Linköping University, Linköping, Sweden; 5 Department of Production Animal Clinical Sciences, Norwegian School of Veterinary Science, Sandnes, Norway; 6 Department of Microbiology and Virology, University of Tromsø, Tromsø, Norway; 7 Present Address: Department of Environmental and Health Studies, Telemark University College, Bø, Norway

**Keywords:** Lyme borreliosis, *Ixodes ricinus*, TaqMan real-time PCR, Norway, *flaB* gene, Prevalence, *Borrelia burgdorferi* sensu lato

## Abstract

A consensus TaqMan real-time PCR test targeting the chromosomal *flaB* gene of *Borrelia burgdorferi* sensu lato was constructed. The test was compared with a recently published generic Light Upon eXtension (LUX) 16S rRNA real-time PCR test (Wilhelmsson et al. in J Clin Microbiol 48:4169–4176, 2010) on material consisting of 242 *Ixodes ricinus* ticks collected from dogs and cats in Northern Norway (n = 139) and Telemark County in Southern Norway (n = 103). Ticks positive in either test were further tested by nested PCR amplification of the 5S-23S rRNA intergenic-spacer region followed by sequencing for species identification. A tick was defined as *Borrelia* positive if two of three tests were positive. Thirty-four of the 242 (14 %) ticks satisfied this definition of positivity. Of these ticks 32 were positive both in the rRNA and *flaB* test, while two were positive only in the rRNA test. One tick was positive only in the rRNA test and was considered false positive since PCR for sequencing failed. The sensitivity of the *flaB* test was 94 % and the specificity 100 %. It was possible to determine the species present using Tm analysis. Among ticks from Northern Norway the prevalence of *Borrelia* was 13 %, whereas the prevalence in Telemark was 16 %. Among identified species (n = 33) *B. afzelii* was found in 16 (47 %), *B. garinii* in 15 (44 %) and *B. valaisiana* in 2 (6 %) ticks, respectively. The *flaB* test is a rapid, sensitive and specific test for detection and quantification of *Borrelia burgdorferi* s.l. in *I. ricinus* ticks. This is the first report on *Borrelia* prevalence in *I. ricinus* in Northern Norway.

## Introduction

Lyme borreliosis (LB) is caused by bacteria of the *Borrelia burgdorferi* sensu lato (s.l.) complex, which infect through the bite of the hard tick *Ixodes ricinus*. *Borrelia burgdorferi* s.l. encompasses eighteen genospecies of which six are associated with human infections (Stanek and Reiter 2011). LB is the most common vector-borne disease in Europe, although showing great regional variation (Stanek and Strle 2003; Stanek and Reiter 2011). In Norway, ticks and LB are abundant along the southern coast. Earlier observations indicate that *I. ricinus* ticks are not endemic north of Brønnøy in Northern Norway (Tambs-Lyche 1943; Braathen et al. 1987). However, recent studies indicate that ticks may be found on dogs and cats further north (Jore et al. 2011, Meldal unpublished material), and they are believed to be imported by migrating birds (Comstedt et al. 2006; Olsen et al. 1995).

In Norway, invasive LB is a notifiable disease, and 250–350 cases are reported yearly to the National Institute of Health, with highest prevalence in the counties of Telemark, Aust-Agder, Vest-Agder and Møre og Romsdal. In 2001 Jenkins et al. found *Borrelia* ssp. in 16 % of the ticks on an island in the southern part of Telemark. A prevalence of 25 % of *B. burgdorferi* s.l. in questing nymphal and adult ticks was recently demonstrated in the southernmost county in Norway (Kjelland et al. 2010).

Recently, several PCR based methods have been developed for analysis of *Borrelia* spp. in clinical samples and ticks (Ivacic et al. 2007; Wilhelmsson et al. 2010). Speed, sensitivity and the possibility of species typing and quantification of *Borrelia* spp. are major advantages of PCR based molecular methods. The introduction of real-time PCR technologies has further reduced analysis times and improved reliability by eliminating the problem of carry-over contamination.

Consensus PCR tests offer an attractive approach for the detection of various *B. burgdorferi* s.l. spp. in ticks. Design of such assays presupposes genes that are ubiquitous in *Borrelia* spp. and well sequenced such as *ospA* (Ivacic et al. 2007) and the 16S rRNA gene (Wilhelmsson et al. 2010).

In this study, we developed and evaluated a consensus TaqMan real-time PCR test targeting a third well sequenced gene, *flaB*, for the detection and quantification of *B. burgdorferi* s.l. The evaluation was performed through analysis of *B. burgdorferi* s.l. present in *I. ricinus* ticks collected from dogs and cats from the northern and southern part of Norway. Results from our developed test were compared with results obtained using a Light Upon eXtension (LUX) 16S rRNA test (Wilhelmsson et al. 2010). Confirmation of PCR results and species determination was performed by sequencing of the 5S-23S rRNA intergenic-spacer (IGS) region (Postic et al. 1994).

## Materials and methods

### Collection of ticks and microscopy

Twenty-three veterinarians in the three northernmost counties in Norway, i.e. Nordland, Troms and Finnmark (from 65°29′N; 12°14′E to 70°12′N; 28°10′E), and five in the south-eastern county Telemark (from 58°53′N; 9°17′E to 59°59′N; 8°43′E), collected ticks from dogs and cats. The ticks were placed in plastic tubes containing 3 ml 70 % ethanol and sent to the microbiology laboratory for further analysis. The collected ticks were examined by light microscopy.

### Preparation of reverse-transcribed total nucleic acid from ticks

Before nucleic acid (NA) extraction ethanol was decanted, 450 μl RLT lysis buffer (Qiagen, Hilden, Germany) containing 1 % vol/vol β-mercaptoethanol was added into the tube. Tubes were then frozen for 1 h at −180 °C (Crampton et al. 1996) and thawed at room temperature. Disruption was performed by bead beating (25 Hz for 2 min) with a 5 mm stainless steel bead in a TissueLyserII instrument (Qiagen), according to the manufacturer’s instructions. After centrifugation at 20,000*g* for 3 min, 400 μl of the supernatant was used for NA extraction. Total NA purification was then performed in a M48 instrument (Qiagen) using the MagAttract RNA Tissue Mini M48 Kit without the DNase step, according to the protocol of the manufacturer, and eluted in 50 μl of the supplied buffer.

Synthesis of cDNA was performed in a PALM PCR cycler (Corbett Research, Australia) using the Illustra Ready-to-Go RT-PCR Beads kit (GE Healthcare Bio-Sciences AB, Stockholm, Sweden). Fifteen μl purified total NA, 2.5 μg random hexamer primers and one bead were incubated in a total volume of 50 μl for 30 min at 42 °C. The enzymatic reaction was stopped at 95 °C for 5 min. cDNA products were stored at −20 °C.

### *Borrelia flaB* PCR

**Fig. 1:**
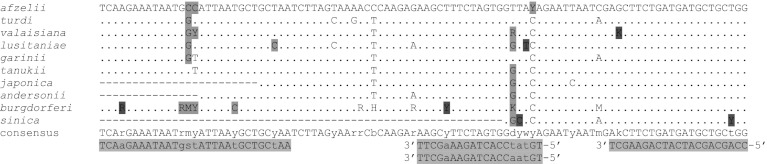
Multiple sequence alignment of target, primer and probe sequences. *Dots* indicated identity to the reference sequence. *Hyphens* indicate regions where no sequence information is available. The following IUPAC ambiguity codes are used: Y = T/C; R = A/G; M = A/C; S = G/C; H = A/C/T; K = G/T; D = A/G/T; B = C/T/G. Variant positions are indicated by *lower case letters* in the consensus and primer/probe sequences. Variants accommodated by G:T basepairing or degeneracy of the primer/probe sequence are highlighted in *green* in the target sequence. Variants not so accommodated are highlighted in *red*. Primer and probe sequences are highlighted in *blue*. Unless otherwise noted, sequences are written 5’ – 3’. (Color figure online)

*Borrelia burgdorferi* s.l. *flaB* sequences showing >90 % homology to *B. valaisiana* DQ111037 were identified by BLAST search and downloaded. After screening out identical sequence replicates CLUSTAL alignments were performed and displayed with BOXSHADE. To keep alignments to a manageable size, the sequences were grouped into four subsets of 50–60 sequences. The resulting consensus sequences were edited manually to reflect the full range of sequence variation and a new alignment using the edited consensus sequences was performed. This led to the identification of a region sufficiently conserved for design of consensus primers. Primer and probe design was performed using Primer Express (Applied Biosystems, Foster City, CA) with *B. burgdorferi* X16833 as input sequence. Variant positions were accommodated by use of the noncanonical G:T basepair, a G/C degeneracy in the forward primer and two probe sequences. Primers, probes and target sequences are shown in Fig. 1 and Table 1. It should be noted that no sequence information for the target region is available for *B. americana, B. bissettii, B. californiensis, B. carolinensis, B. spielmanii, B. kurtzenbachii or B. yangtze* (Stanek and Reiter 2011). Primers were obtained from Genscript Corporation (Piscataway, NJ). TaqMan MGB probes labelled with VIC were obtained from Applied Biosystems. Primer and probe concentrations were optimised leading to the following PCR conditions: 600 nM each primer, 75 nM each probe, 5 μl sample, and Finnzymes DyNAmo Probe qPCR mastermix with ROX (Thermo Fischer Scientific, Vantaa, Finland), reaction volume 25 μl. The PCR program was 50 °C, 2 min; 95 °C, 10 min followed by 40 cycles of 95 °C, 15 s; 60 °C 1 min. For Tm determination the same PCR program, followed by dissociation analysis was performed, using Finnzymes DyNAmo SYBR-green qPCR mastermix (Thermo Fischer Scientific).

**Table 1:** Primer/probe sequences for *flaB* PCR

Name	Function	Sequence	Tm
FlaBf	Forward primer	TCAAGAAATAATGSTATTAATGCTGCTAA^a^	58.8/59
FlaBr	Reverse primer	CCAGCAGCATCATCAGAAGCT	59.2
FlaBmA	Probe	TGTATCCACTAGAAAGCTT	69.5
FlaBm3B	Probe	TGTAACCACTAGAAAGCTT	69.7

^a^S = C + G

To provide control DNA of known concentration, a synthetic plasmid, pFLA, comprised of nucleotides 1–210 from the *B. burgdorferi flaB* sequence X15661 cloned in pUC59 was obtained from Genscript Corporation. This was used to determine analytical sensitivity. Plasmid DNA was serially diluted in a 10 ng/μl solution of purified human genomic DNA (Sigma Aldrich, St Louis, MI) in TE buffer. The limit of detection (Bustin et al. 2009) was determined by testing ten replicates of the lowest and next-lowest detectable dilutions. 10/10 300 GU replicates and 7/10 30 GU replicates were positive. The limit of detection is thus nominally 300 GU.

### LUX 16S rRNA real-time PCR test and species identification

The LUX 16S rRNA real-time PCR test for *Borrelia* spp. analysis was performed according to Wilhelmsson et al. (2010). Briefly, PCR was carried out in 96-well reaction plates (Applied Biosystems), and the 20 μl reaction mixture used contained 10 μl Platinum® qPCR SuperMix UDG (Invitrogen, Carlsbad, CA), 0.04 μl Rox reference (Invitrogen), 0.4 μl LUXTM Bor16SFL primer (10 μM), 0.4 μl unlabeled Bor16SR primer (10 μM) (TIB MOLBIOL, Berlin, Germany), 7.16 μl RNAse free water and 2 μl of template, using an ABI PRISM 7500 Fast Real-Time PCR System (Applied Biosystems). The following reaction conditions were applied, 50 °C for 2 min for the UDG reaction, 95 °C for 2 min for polymerase activation, and then 45 cycles of 95 °C for 15 s, 58 °C for 30 s and 72 °C for 30 s. Melting curve analysis was performed by heating to 95 °C for 15 s, followed by cooling to 60 °C for 1 min and subsequent heating to 95 °C at 0.8 °C per minute with continuous fluorescence recording.

A nested PCR approach was used to amplify the IGS region for species typing, using a PALM PCR cycler (Corbett Research, Australia) (Postic et al. 1994). A final volume of 50 μl reaction mixture, containing 5 μl of 10 × PCR buffer, 1 μl of deoxyribonucleotide triphosphate (10 mM), 1 μl each of the primers 5S-23SF and 5S-23SR (10 μM) (Table 1), 0.38 μl of High Fidelity polymerase (3.5 U/μl) (Amersham Biosciences, Uppsala, Sweden), 5 μl template DNA (2–4 ng/μl) and 36.62 μl of RNase-free water was used. The amplification program consisted of heating at 95 °C for 5 min, followed by 95 °C for 15 s, 57 °C for 30 s, 72 °C for 30 s for 39 cycles and finally 72 °C for 7 min. Five μl of the PCR-product was added to the second PCR reaction mixture with the same volumes, concentrations and amplification programs as the first, using other primers (Bor5S-23SFn and Bor5S-23SRn) and running for 42 cycles (Postic et al. 1994).

Nucleotide sequencing of the PCR-products obtained was performed by GATC (Biotech, Konstanz, Germany). DNA chromatograms were analyzed using the RipSeq web application (iSentio, Bergen, Norway), which allows analysis for a single species as well as mixed samples containing up to three different species (Kommedal et al. 2009).

### Definitions and statistical methods

*Definitions*: *A positive result*: when the sample is positive in both PCR tests, or positive in one PCR test and confirmed by sequencing of the 5S-23S rRNA IGS region. *A negative result*: when negative in both tests, or negative in one PCR and positive in the other, and in addition no PCR product was revealed from the 5S-23S rRNA IGS region.

The kappa statistic was calculated as described in Fleiss et al. (2003).

## Results

All ticks collected were engorged female *I. ricinus*. In total, 242 ticks were collected from dogs (n = 222) and cats (n = 20). Ticks were more abundant in Telemark than in Northern Norway, however, Brønnøy, situated in the southern part of Northern Norway, displayed high tick abundance (33 ticks found on 19 dogs) (Table 2).

**Table 2:** *Borrelia* ssp. positive *Ixodes ricinus* ticks in dogs and cats from veterinary clinics in the county of Telemark and the counties of Northern Norway

Counties	Veterinary clinics, n	Pets, n	Ticks, n	Ticks/pet, n	*Borrelia* positive, n (%)	*B. afzelii*, n (%)	*B. garinii*, n (%)	*B. valaisiana*, n (%)
N. Norway ex. Brønnøy	17	94	106	1.1	10^1^ (9)	5 (50)	4 (40)	0 (0)
Brønnøy	1	19	33	1.7	8 (24)	3 (38)	4 (50)	1 (13)
Telemark	5	71	103	1.5	16 (16)	8 (50)	7 (44)	1 (6)
Totals	23	184	242	1.3	34^1^ (14)	16 (47)	15 (44)	2 (6)

^1^ In one tick, the *Borrelia* genospecies could not be determined

Among 28 dogs that harboured more than one tick, only two dogs had more than one *Borrelia* positive tick (data not shown).

### Evaluation of the PCR assay targeting the *flaB*

A total of 32 ticks were *Borrelia* positive in both real-time PCR tests used, and 207 out of 242 ticks were negative in both tests. Three ticks were only reactive in the rRNA test, kappa = 0.93. All three discrepant ticks had high Cq values (>35), indicative of low concentrations of *Borrelia* NA. In two of these ticks sequencing for species identification was successful. One *Borrelia* reactive tick was considered false positive since sequencing of the 5S-23S rRNA IGS region failed. According to our definitions 34 ticks were therefore considered *Borrelia* spp. positive, whereas one tick showed a false positive result in the rRNA test. Altogether, the rRNA test detected all true *Borrelia* spp. positive ticks, while the newly developed assay failed to detect two positive ticks, reaching a sensitivity of 94 %. The specificity for the developed test was 100 %.

**Fig. 2:**
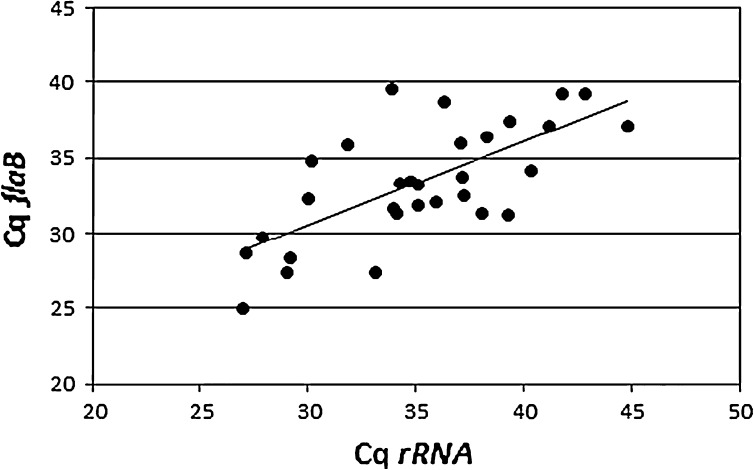
Comparison of Cq values for rRNA-LUX and TaqMan-*flaB* PCR. The slope is y = 0.90x + 4.5 and the correlation coefficient (r^2^) is 0.47

Figure 2 shows a comparison of cycle threshold (Cq) values obtained with the *flaB* and rRNA tests.

### *Borrelia* species and geographical distribution

**Fig. 3:**
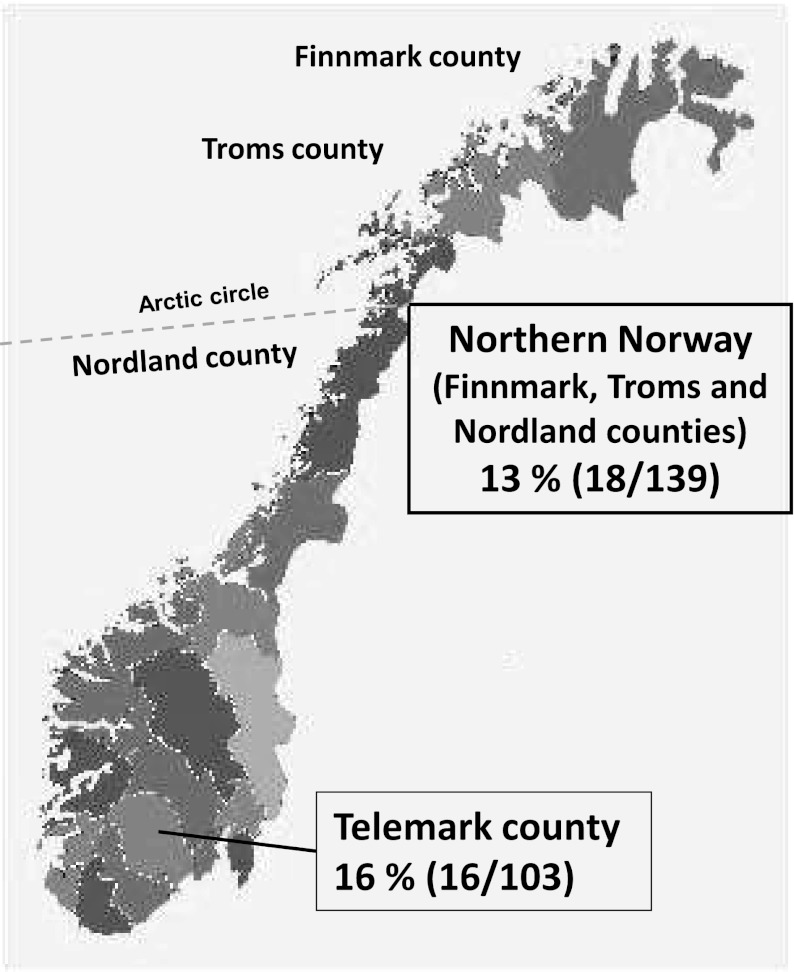
*Borrelia *spp. prevalence in *Ixodes ricinus* ticks in Northern Norway and Telemark

The prevalence of *Borrelia* positive ticks in Telemark was 16 % (16 of 103) and in Northern Norway 13 % (18 of 139), including the municipality of Brønnøy with a prevalence of 24 % (8 of 33) (Table 2 and Fig. 3).

Sequencing (n = 33) of the 5S-23S rRNA IGS region identified *B. afzelii* in 16 (47 %), *B. garinii* in 15 (44 %) and *B. valaisiana* in two (6 %) ticks, respectively. Mixed sequences were not found.

Dissociation analysis of the *flaB* product yielded Tm data for 21 ticks. Ticks containing *B. afzelii* (n = 12), *B. garinii* (n = 8) and *B. valaisiana* (n = 1) showed dissociation peaks at 74.1 ± 0.5, 72.9 ± 0.4 and 77.3 °C, respectively. Nine ticks showed multiple dissociation peaks.

## Discussion

In the present study, we compare our new TaqMan real-time PCR test targeting the *flaB* with a LUX based real-time PCR test targeting the 16S rRNA gene (Wilhelmsson et al. 2010), for detection and quantification of *B. burgdorferi* s.l. in *I. ricinus* ticks. In addition, we present preliminary epidemiological data regarding prevalence and species distribution of *Borrelia* in ticks collected from dogs and cats from different parts of Norway.

The developed test was specific but apparently less sensitive than the rRNA test. The difference in sensitivity is not unexpected as the rRNA PCR test targets a very abundant RNA molecule and is also five PCR cycles longer. In the cases where the *flaB* test gave a false negative result, the rRNA test had a high Cq value, indicative of weakly positive samples. Mismatch to primers or probes is not likely to have caused the discrepancies as neither of the species present, *B. afzelii* and *B. garinii* has sequence variants expected to compromise the sensitivity of flaB PCR.

The rRNA PCR test is a broad-specificity test designed to detect all *Borrelia* spp, including the relapsing fever *Borreliae*. The *flaB* PCR test is designed to detect *B. burgdorferi* s.l. species. On the basis of sequence mismatches (see Fig. 1) the *flaB* test may have reduced sensitivity for *B. sinica*, *B. lusitaniae*, and certain variants of *B. burgdorferi* sensu stricto and *B. valaisiana*, while sequence information for the target region is unavailable for *B. americana*, *B. bissettii*, *B. californiensis*, *B. carolinensis*, *B. kurtzenbachii*, *B. spielmanii*, and *B. yangtze*; and incomplete for *B. japonic*a, *B. andersonii* and *B. sinica*. Of these species, only *B. valaisiana* occurs in the present material. Thus, a more diverse sample material will be required to completely evaluate the *flaB* test. The 48 bp region between the *flaB* primers contains sequence differences which may be revealed by T_m_ analysis. The presence of *B. afzelii*, *B. garinii* and *B. valaisiana* corresponded to melting peaks of 74, 73 and 77 °C, respectively. Hence, our results indicate that Tm analysis of the *flaB* PCR provides a rapid and accurate indication of the *Borrelia* species present, although this has to be assessed on a broader range of *Borrelia* species. In addition, the significance of multiple peaks has to be investigated.

*Borrelia* prevalence in engorged female ticks from Telemark was similar to findings in questing ticks in an earlier investigation from the same county (Jenkins et al. 2001). We had no information from the pet owners whether the ticks were located in close proximity to each other. However, in 28 pets that harboured more than one tick, only two dogs had more than one *Borrelia* positive tick. Hence, co-feeding transmission has not influenced the number of *Borrelia* positive ticks.

Generally, there are more pets with ticks and more ticks per pet seen by the veterinarians in Telemark than in Northern Norway (Table 2).

However, in the municipality of Brønnøy the veterinarian found on average 1.7 ticks per dog, which is even a higher figure than in Telemark. Furthermore, a high proportion (24 %) of the included ticks in Brønnøy were positive for *Borrelia* spp. (Table 2).

In the present study, *B. afzelii*, *B. garinii* and *B. valaisiana* were found in 47 %, 44 % and 6 % of ticks respectively. This distribution differ from recent data including ticks that have bitten humans in Sweden, where *B. afzelii* was present in over 60 % and *B. garinii* in 23 %, respectively (Wilhelmsson et al. 2010). However, our data follow the same *Borrelia* spp. pattern demonstrated in questing adults and nymphal ticks in South Norway (Kjelland et al. 2010).

This is the first report investigating *Borrelia* in ticks collected in Northern Norway. Future studies are needed, using strict criteria regarding for instance stays outside the county of residence, and a larger population of ticks collected from pets, for a better understanding of the *Borrelia* epidemiology in different parts of Norway.

In summary, we have developed a robust, sensitive, specific and rapid PCR test based on *flaB* for detection and quantification of *B. burgdorferi* s.l. with the possibility of rapid species subtyping. Our data give new insights into Norwegian *Borrelia* epidemiology.
